# Knowledge, Beliefs/Attitudes, and Practices of Rural Residents in the Prevention and Control of COVID-19: An Online Questionnaire Survey

**DOI:** 10.4269/ajtmh.20-0314

**Published:** 2020-10-27

**Authors:** Lihua Ma, Hui Liu, Zhang Tao, Ning Jiang, Song Wang, Xiaolian Jiang

**Affiliations:** 1 Institute of Emergency Management and Reconstruction in Post-Disaster, Sichuan University, Chengdu, People’s Republic of China;; 2 The First Hospital of Lanzhou University, Lanzhou, People’s Republic of China;; 3 West China Second University Hospital, Sichuan University, Chengdu, China;; 4 Department of Spine Surgery, The 940 Hospital of Joint Logistics Support Force of Chinese People’s Liberation Army, Lanzhou, People’s Republic of China;; 5 West China Hospital/West China School of Nursing, Sichuan University, Chengd, China

## Abstract

The outbreak of COVID-19 quickly spread to 184 countries and regions around the world. It has drawn great attention from the WHO and was declared an international public health emergency on January 31, 2020. Because the population is generally susceptible to the virus, there are no effective drugs and vaccines, and active participation of the entire population in self-protection and self-isolation has become the key to cutting off transmission routes and effectively controlling the epidemic. A self-designed questionnaire to assess residents’ knowledge, attitudes, and behaviors related to COVID-19 prevention and control used the Questionnaire Star service platform, and snowball sampling was used to invite rural residents to complete the questionnaire on WeChat. A total of 554 valid questionnaires were collected. Rural residents’ average scores on knowledge, attitudes, and behaviors regarding prevention and control were 40 ± 7 (total of 50 points), 45 ± 3 (total of 52 points), and 92 ± 12 (total of 127 points), respectively. A lack of protective materials and weak awareness of prevention and control are the greatest difficulties and challenges experienced by rural residents during the epidemic. Accordingly, social support services, such as public transportation plans, supply chains for living materials, and orderly returns to work, need to be strengthened. Moreover, new infectious disease control is not only a task for individuals but also a global issue. It is of great significance to guarantee information transparency and enhance health risk communication.

## INTRODUCTION

Since the outbreak of COVID-19 in Wuhan, China, in December 2019, it has spread rapidly to 34 provinces, municipalities, and autonomous regions in China and to more than 200 countries around the world; furthermore, it is spreading at an increasing rate (on average, the number of people infected doubles every 3 days).^1^ As of July 4, 2020, the cumulative number of reported confirmed cases of COVID-19 in China exceeded 85,284, and the cumulative number of confirmed cases overseas was 11,130,085.^2^ The WHO declared the virus an international public health emergency on January 31.

According to China’s health statistics yearbook, the construction of health service institutions included 68.84 beds per 10,000 people in cities and 31.14 in rural areas. Per capita health expenditure was 2,969.01 yuan in urban areas and 1,055.89 yuan in rural areas. There is a wide gap in benefits enjoyed by urban and rural residents. With respect to human resources in health care, there are 85 urban health technicians per 10,000 people, which is 2.5 times as many as there are rural health technicians per 10,000 people. The number of urban (assistant) doctors per 10,000 people was 2.3 times the number of rural (assistant) doctors per 10,000 people. The number of registered nurses working in city and county hospitals was 3.6 times that in rural township hospitals. The start of the Chinese Spring Festival is one of the most celebrated times of the year and each Chinese coincides with the emergence of COVID-19. During this period, mass migration as individuals return home. It is estimated that nearly three billion trips will be made during the 40-day travel rush. Before the travel ban began on January 23, 2020, about 5 million people left Wuhan (the capital of Hubei Province), the epicenter of the COVID-19 epidemic.^3^ About 60% of these people went to rural areas outside Hubei Province. Limiting these people’s social contact is critical to controlling COVID-19, as problems in China’s rural areas, including low economic level, poor awareness of disease response, and inadequate environmental controls (sewage treatment, household waste separation, and toilet use), make COVID-19 more difficult to control.^3–5^

However, the allocation of rural health resources at the grassroots level in developed areas should strive to improve the efficiency of the use of health resources and provide effective medical and health services.

People are generally susceptible to the virus, and there are no effective drugs or vaccines. Controlling the source of infection and cutting off the transmission route have become the only reliable and directly effective epidemic control measures. Whether transmission can be blocked depends on the active participation of the people, high levels of self-discipline, and effective adoption of self-defense behavior. According to the China Demographic Network, China has 564,010,000 rural residents, accounting for 40.4% of the total population. Rural residents have the characteristics of relatively scattered living areas, limited social connections, and a lack of medical resources. Furthermore, the outbreak of COVID-19 and the epidemic peaked during the return and resumption of work during and after the Spring Festival. Rural residents constitute the largest mobile population at 60%, which greatly increases their susceptibility to disease and the risk of infection.^6^ Rural residents have no directionality of movement, and they will go anywhere close to home for health care. Trains and villages as the two gathering places of temporarily migrating populations during the Spring Festival. Both places are more vulnerable and provide ideal conditions for the cross-transmission of the virus because of the impact of the environment and protective measures. Rural residents account for the largest number of people returning to the Spring Festival. Rural areas have become an important battlefield for epidemic prevention and control after residents return home. At the same time, compared with urban residents, rural residents have (a lower education level and delete) relatively weak awareness of disease prevention and control; because of their remote residence and poor economic conditions, the availability of protective materials, protective facilities, health education, and medical services is lower than that of cities. Furthermore, there is a large gap between residents. These factors have greatly increased the difficulty of preventing and controlling epidemics in rural areas. To this end, this study uses an online survey to understand rural residents’ knowledge, attitudes, behaviors, and other factors related to COVID-19 prevention and control as well as the difficulties and challenges they experience in the process of self-protection. To improve residents’ capabilities and behaviors and optimize epidemic management decision-making by the government, this study suggests targeted interventions for epidemic prevention and control.

## OBJECTIVE

The aims of this study were to investigate the knowledge, attitudes, and behaviors related to the prevention and control of COVID-19 among rural residents; to analyze the influencing factors, difficulties, and challenges of prevention and control in this population; and to develop a plan to improve rural residents’ awareness of COVID-19 prevention and control, for optimizing the epidemic management decision-making by the government.

## METHODS

### Survey tools.

(1) General information questionnaire: gender, age, education level, marital status, whether you have experienced SARS, etc.
(2) Questionnaire on knowledge, attitude, and behavior of COVID-19: Based on the “*Knowledge, Attitude/Belief, and Practice*” (KAP) model,^7–9^ with reference to the COVID-19 Prevention and Control Guidelines issued by the WHO. The questionnaire on knowledge, attitude, and behavior of COVID-19 refers to the consensus of Chinese experts, such as the Chinese Health Commission and the CDC, as well as the prevention and control knowledge provided by official public platforms, popular education documents, and related research literature. The knowledge content includes 11 aspects, namely, the classification of infectious diseases, the source of infection, the transmission route, etc. (refer the prevention and control knowledge of COVID-19 Supplemental Questionnaire); sources of knowledge: including WeChat, online news, television, etc. Prevention/control/attitude refers five items and 13 aspects: including awareness of the necessity of prevention and control, perception of the possibility of being infected, willingness to take prevention and control measures, etc. Each item was rated on a Likert scale, four or five hierarchy was usually used. The item scores of prevention/control/attitude was used as four hierarchy. Unnecessary is one point, somewhat necessary is two points, necessary is three points, very necessary is four points, and the total score ranged from 13 to 52 points. Practice of prevention and control includes daily living hygiene, diet, exercise, and sleep behavior, yielding a total of 27 items. Except for two dichotomous items, other items were rated on a five-point Likert scale. Never is one point, seldom is two points, sometimes is three points, usually is four points, always is five points, and the total score ranged from 54 to 121 points. Open-ended question: what difficulties and challenges have you encountered in the prevention and control of the epidemic? The Questionnaire Star (https://www.wjx.cn/) online survey platform was used to record the questionnaire, forming an online questionnaire. However, its power of transmission is limited. In other words, its information is limited to the platform on which the information resides. During the COVID-19 pandemic, everyone was quarantined at home and had plenty of time to complete the questionnaire. Therefore, this would not be a burden on the respondent in terms of time and connection costs.

### Survey methods and objects.

A “snowball sampling” method was used in each province. First, a group of rural residents was selected at random. Second, these rural respondents were interviewed and asked to provide the names of other respondents in the targeted rural residential population. According to the clues provided, the respondents were selected. This process was continued, which created a snowball effect. Although random sampling was used to select the respondents initially, the final samples were all nonprobability samples.

From February 5, 2020 to February 13, 2020, the epidemic is rising. Based on the questionnaire star service platform, using descriptive research and snowball sampling methods to invite rural residents to participate in the survey, residents use mobile phone WeChat to fill in online. The term “rural residents” refers to residents whose household registration is with the villagers’ committee and who are held responsible for the land independently or through their parents. Official functionaries working in state organs, institutions, or people’s organizations who have worked in enterprises for at least three consecutive years enjoyed the social security benefits of urban residents; these individuals have purchased houses in cities and towns for at least three consecutive years but have not moved their household registration to the residents’ committee and are regarded as urban residents (noted and explained in the questionnaire). The survey involved a total of 12 basic information, 76 questionnaire items, 18 dimensions, and 30 variables for statistical analysis.^10^ According to the Kendall sample estimation method of multivariate analysis, the number of samples required should be 10–20 times the number of variables. The minimum sample size for this survey was calculated to be 300–600. The subjects invited to participate in the survey are rural residents older than 14 years, living in rural areas during the survey period, and willing to voluntarily participate after informed consent. A total of 554 online questionnaires were collected. To assess the present situation of rural residents’ COVID-19 control and the reliability and validity of the questionnaire comprising three scales (knowledge, attitudes, and behaviors), 60 local villagers were chosen to complete the questionnaire. The results show that the Cronbach’s α coefficient for the three subscales (knowledge, attitudes, and behaviors) was 0.884, 0.869, and 0.821. The Spearman-Brown split-half coefficients were 0.827, 0.834, and 0.807. The reliability measured 2 weeks after the first attempt with the same sample was 0.851.

### Data analysis.

The SPSS software (version 25.0; SPSS Inc., Chicago, IL) was used for statistical analysis in this study. Continuous variables were described as means with SDs, whereas categorical variables were presented as frequencies with percentages. The item scoring rate and the total scoring rate for KAP were calculated by dividing the actual score of an item or total items by the total item/items score and multiplying by 100%. Two independent samples *t*-tests or one-way analysis of variance (ANOVA) were conducted to evaluate the differences among respondents with different social demographic characteristics. Pearson correlation analysis was carried out to examine the relationships among KAP. The statistically significant variables identified in univariate analysis, and those professionally considered as the significant factors, were screened as the independent variables, which were incorporated into the multivariate linear regression analysis equation to further clarify the influencing factors of KAP. A difference of *P* < 0.05 was considered as statistically significant (two-tailed).

## RESULTS

### Demographic characteristics.

A total of 559 questionnaires were recovered in this survey, and each questionnaire item was set up for mandatory responses. Otherwise, the questionnaires could not be submitted, and only completed questionnaires could be received. Five invalid questionnaires were eliminated. Two of them had the same IP address (one resident may have completed two questionnaires), and three of the respondents disagreed with scientific research. Thus, a total of 554 valid questionnaires were collected. The effective questionnaire recovery rate was 99.1%. The basic characteristics of the respondents are shown in Table 1.

**Table 1:** The characteristics of the respondents

Variable		*n* (%)

Gender	Female	342 (61.7)
	Male	212 (38.3)
Age (years)	≤ 30	266 (48.1)
	31–45	188 (33.9)
	≥ 46	100 (18.0)
Nationality	Han	529 (95.5)
	Others	25 (4.5)
Education level	Undergraduate or associate degree	79 (14.3)
	Senior high school or Vocational degree	232 (41.9)
	Junior school and below	243 (43.8)
Marital status	Married	241 (43.5)
	Unmarried/Divorced/Widowed	313 (56.5)
Occupation	Farmer	125 (22.5)
	Enterprise workers	153 (27.6)
	Student	239 (43.1)
	Others	37 (3.0)
Family economic level	Good	15 (2.7)
	Medium	377 (68.1)
	Poor	162 (29.2)
With chronic disease or not	No	535 (96.6)
	Yes	19 (3.4)
With confirmed cases in your residential area or not	No	400 (72.2)
	Yes	69 (12.5)
	Unknown	85 (15.3)
Have been to the epidemic area (such as Hubei Province of China) or not	No	547 (98.7)
	Yes	7 (1.3)
Have experienced SARS or not	No	452 (81.6)
	Yes	102 (18.4)

The survey objects of this study were rural residents in various provinces with wide coverage. The respondents included women, people of Han nationality, those with a junior high school education and below, unmarried/divorced/widowed people, farmers, people with middle-level family income, those without chronic diseases, people with no confirmed cases in their residential area, not having been to an epidemic area (such as Hubei Province in China), and having experienced the SARS epidemic. The areas were mainly related to the place where the questionnaire respondents were located or their schools. The average age of the rural residents in this study was 30.05 years old. Enterprise workers and students accounted for 70% of the total rural residents in this survey and 60% of returnees during the Spring Festival. However, during the epidemic, the state placed restrictions on personnel flow, resumed work, and resumed production.^11^ Requirements such as delays in spring plowing and home isolation for at least 14 days have a definite impact on the social economy, and enterprise workers account for more than 60% of labor-intensive industries and enterprises such as manufacturing, construction, and service industries. Students older than 14 years are usually high school/secondary school/university/graduate students. They have the characteristics of highly concentrated personnel, extensive social connections, and collective activities. After the winter vacation, students will return to the university, and the university will soon become the main battlefield for epidemic prevention and control. Students are also one of the most vulnerable groups.^12^ Once the epidemic spreads in schools, it will affect the stability of families, schools, and society. Therefore, relevant research on the impact of the epidemic on the industry should be considered, and corresponding countermeasures should be implemented.

#### Knowledge, attitude, and behavioral scores of rural residents with regard to COVID-19.

**Figure 1.**
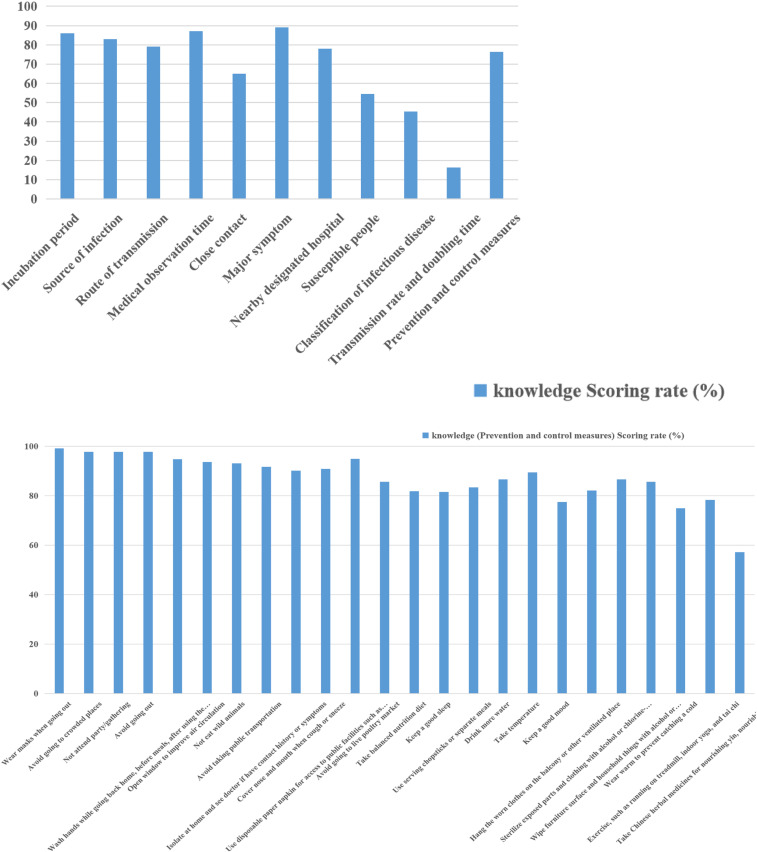
Knowledge scoring rate (%). Knowledge (prevention and control measures) scoring rate (%) (Supplemental Table 1). This figure appears in color at www.ajtmh.org.

**Figure 2.**
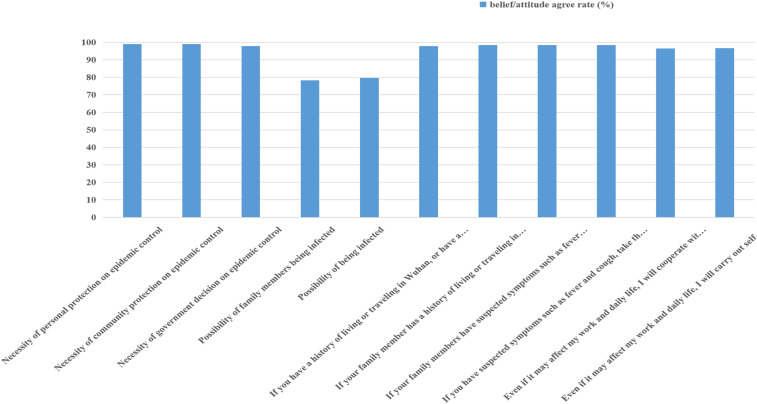
Belief/attitude agree rate (%) (Supplemental Table 2). This figure appears in color at www.ajtmh.org.

**Figure 3.**
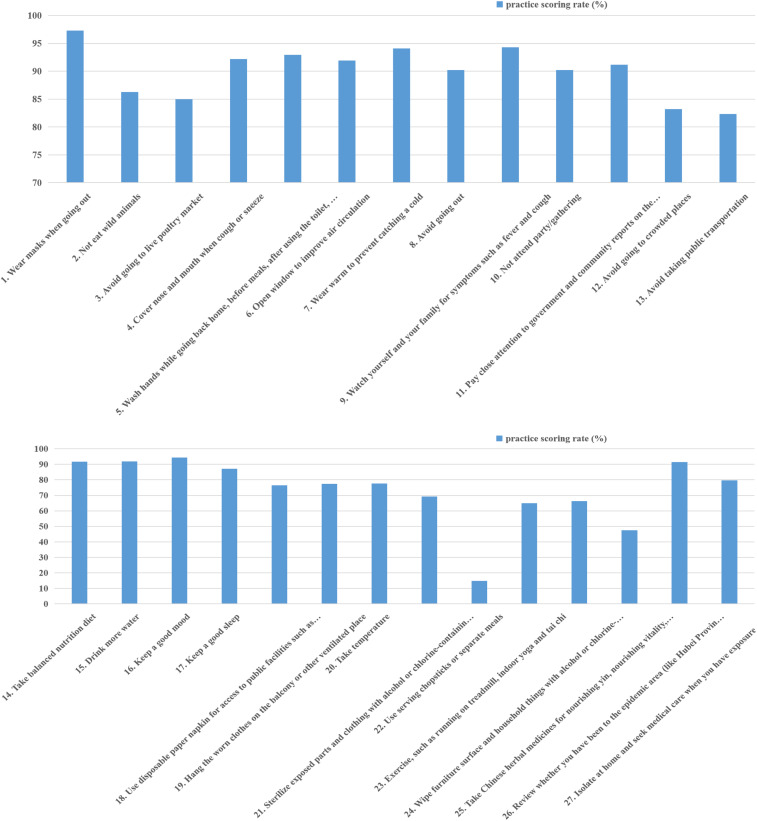
Practice scoring rate (%) (Supplemental Table 3). Score rate (%) of residents’ practice on prevention and control of COVID-19 = the number who sometimes or often or always have done/*N* (554). This figure appears in color at www.ajtmh.org.

The overall average knowledge score was 39.75 ± 6.703, the total scoring rate was 65.5%, the highest item scoring rate was 87.2% (close observer observation time), and the lowest was 16.2% (spread rate). The overall belief/attitude score was 45.40 ± 3.341, the total score was 84.7%, the highest score was 98.9% (the need for personal and community protection to control the epidemic), and the lowest was 78.3% (the awareness of the possibility of being infected). The average behavior score was 104.69 ± 12.167, the total scoring rate was 65.3%, the highest item score was 97.3% (wear a mask when going out), and the lowest was 14.8% (eating with chopsticks or eating at different meals). The results are shown in Tables 2–4 and Figures 1–3.

**Table 2:** Scores of residents’ knowledge on prevention and control of COVID-19 (*N* = 554)

Item	Score range	$\bar{x}$ ± SD
Incubation period	0–1	0.86 ± 0.347
Source of infection	0–3	2.78 ± 0.514
Route of transmission	0–3	2.76 ± 0.503
Medical observation time	0–1	0.87 ± 0.335
Close contact	0–3	2.51 ± 0.732
Major symptom	0–5	4.61 ± 1.041
Nearby designated hospital	0–2	1.36 ± 0.479
Susceptible people	0–5	3.44 ± 1.410
Classification of infectious disease	0–1	0.45 ± 0.498
Transmission rate and doubling time	0–2	0.72 ± 0.726
Prevention and control measures	0–24	20.85 ± 4.716
Wear masks when going out	0–1	0.99 ± 0.095
Avoid going to crowded places	0–1	0.98 ± 0.152
Not attend party/gathering	0–1	0.98 ± 0.146
Avoid going out	0–1	0.98 ± 0.152
Wash hands while going back home, before meals, after using the toilet, or contacting with dirty and contaminated items	0–1	0.94 ± 0.244
Open window to improve air circulation	0–1	0.95 ± 0.227
Not eat wild animals	0–1	0.93 ± 0.253
Avoid taking public transportation	0–1	0.92 ± 0.276
Isolate at home and see doctor if have contact history or symptoms	0–1	0.90 ± 0.299
Cover nose and mouth when cough or sneeze	0–1	0.91 ± 0.289
Use disposable paper napkin for access to public facilities such as elevator buttons, and door handles	0–1	0.87 ± 0.333
Avoid going to live poultry market	0–1	0.86 ± 0.350
Take balanced nutrition diet	0–1	0.82 ± 0.385
Keep a good sleep	0–1	0.82 ± 0.388
Use serving chopsticks or separate meals	0–1	0.83 ± 0.372
Drink more water	0–1	0.87 ± 0.341
Take temperature	0–1	0.90 ± 0.306
Keep a good mood	0–1	0.77 ± 0.418
Hang the worn clothes on the balcony or other ventilated place	0–1	0.82 ± 0.383
Sterilize exposed parts and clothing with alcohol or chlorine-containing disinfectant when returning home	0–1	0.87 ± 0.341
Wipe furniture surface and household things with alcohol or chlorine-containing disinfectant	0–1	0.86 ± 0.352
Wear warm to prevent catching a cold	0–1	0.75 ± 0.434
Exercise, such as running on treadmill, indoor yoga, and tai chi	0–1	0.78 ± 0.412
Take Chinese herbal medicines for nourishing yin, nourishing vitality, tonifying spleen, and moistening lung	0–1	0.57 ± 0.495

Score rate (%) of residents’ knowledge on prevention and control of COVID-19 = the number of someone who answer right/N (554).

**Table 3:** Scores of residents’ belief/attitude on prevention and control of COVID-19 (*N* = 554)

Dimension	Item	Score range	$\bar{x}$ ± SD
Necessity of prevention and control	Necessity of personal protection on epidemic control	1–4	3.89 ± 0.360
	Necessity of community protection on epidemic control	1–4	387 ± 0.377
	Necessity of government decision on epidemic control	1–4	3.84 ± 0.449
Possibility of being infected	Possibility of family members being infected	1–4	3.15 ± 0.931
	Possibility of being infected	1–4	3.18 ± 0.886
Willingness to take prevention and control measures	If you have a history of living or traveling in Wuhan, or have a history of close contact with an infected person, take the initiative to report and isolate at home	1–4	3.71 ± 0.516
	If your family member has a history of living or traveling in Wuhan, or have a history of close contact with an infected person, persuade them to take the initiative to report and isolate at home	1–4	3.69 ± 0.502
	If your family members have suspected symptoms such as fever and cough, persuade them to take the initiative to isolate themselves and go to see doctor in fever clinic	1–4	3.69 ± 0.502
	If you have suspected symptoms such as fever and cough, take the initiative to isolate at home and go to see doctor in fever clinic	1–4	3.69 ± 0.502
	Even if it may affect my work and daily life, I will cooperate with the government and community for epidemic prevention and control	1–4	3.62 ± 0.569
	Even if it may affect my work and daily life, I will carry out self-protection measures	1–4	3.61 ± 0.577
Attitude toward eating wild animals	–	1–4	3.91 ± 0.329
Attitude toward legislation to prohibit the eating of wild animals	–	1–4	3.79 ± 0.557

**Table 4:** Scores of residents’ practice on prevention and control of COVID-19 (*N* = 554)

Item	Score range	$\bar{x}$ ± SD
1. Wear masks when going out	1–5	4.77 ± 0.521
2. Not eat wild animals	1–5	4.48 ± 1.091
3. Avoid going to live poultry market	1–5	4.43 ± 1.087
4. Cover nose and mouth when cough or sneeze	1–5	4.56 ± 0.782
5. Wash hands while going back home, before meals, after using the toilet, or contacting with dirty and contaminated items	1–5	4.58 ± 0.676
6. Open window to improve air circulation	1–5	4.55 ± 0.685
7. Wear warm to prevent catching a cold	1–5	4.56 ± 0.637
8. Avoid going out	1–5	4.58 ± 0.752
9. Watch yourself and your family for symptoms such as fever and cough	1–5	4.59 ± 0.639
10. Not attend party/gathering	1–5	4.58 ± 0.752
11. Pay close attention to government and community reports on the epidemic and the living trajectory of infected people	1–5	4.52 ± 0.714
12. Avoid going to crowded places	1–5	4.37 ± 1.139
13. Avoid taking public transportation	1–5	4.34 ± 1.151
14. Take balanced nutrition diet	1–5	4.53 ± 0.724
15. Drink more water	1–5	4.55 ± 0.682
16. Keep a good mood	1–5	4.59 ± 0.639
17. Keep a good sleep	1–5	4.40 ± 0.772
18. Use disposable paper napkin for access to public facilities such as elevator buttons and door handles	1–5	4.41 ± 1.197
19. Hang the worn clothes on the balcony or other ventilated place	1–5	4.22 ± 0.972
20. Take temperature	1–5	4.26 ± 0.913
21. Sterilize exposed parts and clothing with alcohol or chlorine-containing disinfectant when returning home	1–5	3.26 ± 1.074
22. Use serving chopsticks or separate meals	1–5	2.04 ± 1.225
23. Exercise, such as running on treadmill, indoor yoga and tai chi	1–5	4.00 ± 1.093
24. Wipe furniture surface and household things with alcohol or chlorine-containing disinfectant	1–5	4.94 ± 1.165
25. Take Chinese herbal medicines for nourishing yin, nourishing vitality, tonifying spleen, and moistening lung	1–5	3.47 ± 1.339
26. Review whether you have been to the epidemic area (such as Hubei Province of China) during the epidemic, or have contact with infected people	0–1	0.92 ± 0.279
27. Isolate at home and seek medical care when you have exposure or symptoms such as fever and cough.	0–1	0.80 ± 0.403

#### Univariate and multivariate analyses of factors related to KAP scores.

According to the results of two independent-sample t-tests and one-way ANOVA, there were significant differences in the knowledge scores between respondents with different genders, education levels, occupations, family economic levels, incidence of chronic diseases, presence or absence of confirmed cases in their residential villages, and not experienced of SARS. There were significant differences in the attitude scores between respondents with different genders, ages, education levels, occupations, and experience of SARS. There were significant differences in the behavioral scores between respondents with different genders, ages, education levels, occupations, family economic levels, incidence of chronic diseases, presence or absence of confirmed cases in their villages and towns, and experience of SARS.

Correlation analysis showed that the scores of knowledge, attitude, and behavior were positively correlated, and the correlation between attitude and behavior was the strongest (correlation coefficient *r* = 0.423) (*P* < 0.001).

Multiple linear regression analysis showed that residents with an education level of junior high school or below and occupations as farmers had significantly lower scores in knowledge, attitude, and behavior than those with high school/secondary school, college or higher education (*P* < 0.05); the knowledge and behavior scores of families with poor economic conditions were significantly lower than those with good and moderate family economic conditions (*P* < 0.05); and residents with chronic diseases and those living in areas with confirmed cases had significantly higher knowledge and behavior scores than those without chronic diseases and no or unknown confirmed cases in their area (*P* < 0.05).

### Information sources and the possible difficulties and challenges encountered.

The survey showed that there are 10 types of information sources for rural residents to receive information on prevention and control. Six of them have a utilization rate of more than 50%, including WeChat (89.2%), Internet news (87.2%) and TV (85.0%), community/village epidemic prevention propaganda (75.6%), government announcements (69.7%), and short messages (60.1%). The results are shown in Table 5.

**Table 5:** Information sources of residents’ knowledge on prevention and control of COVID-19 (*N* = 554)

Source	*n* (%)
WeChat	494 (89.2)
Network news	483 (87.2)
TV	471 (85.0)
Community/village epidemic prevention pamphlet/bulletin board/campaign	419 (75.6)
Government announcements	386 (69.7)
SMS	333 (60.1)
Radio	267 (48.2)
Work unit	11 (2.0)
Micro-blog	5 (0.9)
Others (informed by others, Douyin app, etc).	13 (2.3)

A total of 401 people received feedback on self-control difficulties and challenges, accounting for 72.4% of the total. Rural residents reported difficulties and challenges in four main areas (representing more than 10% of the total number of responses): lack of protective equipment (40.2%), inconvenience of travel (23.7%), lack of awareness of prevention and control (16.9%), and life inconvenience (10.7%). The results are shown in Table 6.

**Table 6:** Difficulties and challenges encountered by residents in epidemic prevention and control (*N* = 401)

View	*n* (%)
1. Protective equipment: rural residents, who especially resided in remote mountainous areas, cannot buy masks, alcohol, disinfectant, gloves, etc. and rural medical workers/traffic police/duty personnel at the village entrance do not have protective equipment such as isolation clothing and goggles.	161 (40.15)
2. Inconvenience of going out: suspension of public transport, duty at the entrance of the village and banned in and access, and return work unit difficult	95 (23.69)
3. Weak awareness of prevention and control: patients or people in incubation period conceal their condition, old people do not wear masks gathered in the sun, epidemic period visit, playing mahjong, and the family does not wash their hands in time after going out	68 (16.96)
4. Inconvenience to buy daily necessities: vegetables, rice, flour, oil, and baby products	43 (10.72)
5. Affecting study and work: school delay, affecting study, worry about insufficient prevention and control conditions, lack of necessary prevention and control facilities, and cross infection at work units	31 (7.73)
6. Affecting psychology: boredom arises from prolonged isolation at home, feeling nervous and panic when seeing the epidemic related reports and patient pictures, and fear of being infected with COVID-19 whenever there is any discomfort present	23 (5.73)
7. Economic damage: delayed farming, crops (apples and shiitake) cannot be shipped out, and vegetable prices double	16 (3.49)
8. Information reliability: lack of access to truthful and reliable information about the epidemic situation; inability to distinguish between common cold and COVID-19; unclear treatment of protective equipment such as contaminated masks	15 (3.74)

## DISCUSSION

### Rural residents’ overall KAP of the prevention and control of COVID-19.

According to our research results, the score rate for KAP were 65.5%, 84.7%, and 65.3%, respectively. The overall knowledge and behavior of rural residents are at a medium level, and attitudes are at a high level. These achievements are related to the new edition of the COVID-19 Education Manual issued by the government, health committees, disease control centers, and university hospitals and to the publicity, education, and supervision of communities, villages, and the media.^13^ Effective risk communication in the early stages of infectious diseases, timely understanding of relevant knowledge, and attitudes and behaviors of the public are very important to reduce the negative and panic mentality caused by the epidemic, adopt targeted health education strategies and measures, and effectively prevent and control the spread of disease. This epidemiological study conducted a rapid assessment of rural residents’ knowledge, beliefs, and actions during the rising stage of the new corona pneumonia epidemic, which can provide a basis for the government to develop targeted health education and behavioral intervention strategies.

### Analysis of COVID-19 knowledge, belief/attitude, behavioral items, and influencing factors among rural residents.

#### Knowledge.

This survey shows that the source of COVID-19 infection, the route of transmission, incubation period, main symptoms, time of close contact isolation and observation, and awareness rate of personal protective measures are above 70%, and the score rate of 20 of the 24 protective measures is above 80%, indicating that most rural residents have better knowledge of new crown pneumonia prevention and control, which is inseparable from government departments’ propaganda and education for villagers. The scoring rates for new corona pneumonia infectious diseases, susceptible populations, and transmission rates are below 60%, at 45.3%, 54.6%, and 16.2%, respectively (see Table 2 and Figure 1). The reasons for these findings may be that this COVID-19 knowledge is highly specialized, and through investigation, rural residents often cannot obtain COVID-19 knowledge through simple WeChat and news information. Instead, it is necessary to develop rural publicity and education methods based on the characteristics of rural populations (see Table 5).

Regression analysis shows that men’s knowledge scores are more different than women’s knowledge scores. Possible reasons are that men’s information processing speed and execution ability are generally more different than that of women. Because of the influence of traditional ideas (rural masculinism), rural women mainly undertake housework and have a relatively low education level.^14^ Some females have dropped out of junior high school and have poor knowledge of COVID-19; those with a junior high school education or below with a knowledge score lower than that of junior high school may be subject to this level of education. COVID-19 knowledge is limited in its grasp and understanding. WeChat and related news and information alone cannot provide relevant knowledge and it is best to have professional guidance.

Rural residents with poor household economic scores have lower knowledge scores than those with medium and high economic conditions. Residents with poor economic conditions may not pay much attention to health care and may not have the motivation to actively master knowledge.^15^

Rural residents with chronic disease have less information than those without. High knowledge scores among residents may be related to previous research reports (40% of patients with COVID-19 died of chronic diseases). People with chronic diseases experienced preventive treatment of the disease in the early stage and were more concerned about their own body changes.

Of the rural residents, 81.5% had experienced SARS, and rural residents who had experienced SARS had higher knowledge scores than those who had not experienced SARS. The possible reasons are that SARS is similar to COVID-19, and both are infectious diseases of the respiratory tract.

The prevention and control measures are basically the same, so it is necessary to conduct timely drills and multi-department cooperation to allow residents to obtain epidemic-related knowledge, effectively improve the level of epidemic prevention and control of residents in rural communities, and provide security for rural residents who experience public health emergencies. Prevention and control provide effective methods. It is suggested that in the promotion of knowledge about the prevention and control of new coronary pneumonia, women, farmers, families with poor economic conditions, people with no chronic diseases, those with no confirmed cases in their villages and towns, and people who have not experienced SARS should be the key educational objects.

The analysis of information sources shows that the main way for rural residents to obtain prevention and control knowledge is the mass media, indicating that mainstream media (such as WeChat, online news platforms, and television) play a very important role in disseminating prevention and control knowledge.^16^ Studies have shown that the integrity of the early warning system and the public’s timely access to information will directly affect the ability to respond to public health emergencies. Therefore, it is necessary to further strengthen the construction of modern communication network infrastructure and give full play to the active role of mainstream media in the process of disseminating news on public events and health education so that information is timely, accurate, scientific, and accessible.

### Belief/attitude.

Seventy-eight percent of rural residents think that they and their family members may be infected. The main reasons may be the high contagion level of COVID-19 and the lack of effective treatment methods.^17^ This finding suggests that in the rising stage of the disease epidemic, timely and accurate transmission of key information to rural areas is very important to help the public through the crisis. We should continue to organize corresponding health education and publicity in a timely manner and address the concerns of rural residents according to the latest epidemic situation. More than 97% of the respondents believe that personal protection, community protection, and government decision-making are necessary for epidemic control and are willing to cooperate with the community and government’s prevention and control work (see Table 3 and Figure 2).^18,19^ This may be related to the Chinese government’s high emphasis on the prevention and control of new coronary pneumonia and the restrictions imposed, including a series of measures related to personal travel in key areas and social mobilization. A total of 98.6% of residents’ held the attitude toward edible game that they “do not eat it themselves, and they are also opposed to eating by others.” Of rural residents, 97.1% agree that the country currently has a law prohibiting the hunting, buying, and selling of wild animals, which may be related to the source of the epidemic. With regard to wild animals, rural residents have a more positive attitude toward prevention and control and a stronger sense of social responsibility. This is also one of the important factors in controlling the epidemic. After 2 months of prevention and control cooperation, newly diagnosed cases of new coronary pneumonia in rural residents have basically not increased.^20^ This phenomenon is inseparable from the efforts of rural individuals, communities, and the government and is a positive reflection of qi and other public attitudes about new coronary pneumonia. The results are consistent. The analysis also shows that even though 98.7% of the respondents in this survey were residents of severely affected areas (Hubei Province), only 21.7% believed that they could not be infected, and 20.3% believed that their families could not be infected. This shows that village residents have a good perception of the danger of the epidemic; however, this may also have a certain impact on the public’s psychology and cause panic, which needs to be further explored.

Regression analysis showed that gender, age, economic level, and experience of SARS are predictors of beliefs/attitudes. Higher information levels among males, those aged 30 years and younger, and experience with the SARS epidemic indicate that the SARS epidemic response experience has a positive effect on confidence in epidemic prevention and control. Female residents who are older than 30 years and in poor economic condition should be the focus group for epidemic prevention and control.

### Behavior.

An analysis of the items showed that 18 of the 27 behaviors had scores above 80% (see Table 4 and Figure 3). Low-scoring items indicated that the implementation of infrequent prevention and control measures needs to be further strengthened, such as “eating with chopsticks or splitting meals” and “taking Chinese herbal medicine ingredients for yin, qi, spleen, and lungs.” Not using public chopsticks for meals may be related to the constraints of traditional Chinese concepts. Many people still think that using public chopsticks is a manifestation of distrust and mutual disapproval among people, especially when eating with family members.^20^ The Municipal Health Promotion Committee and the Office of the Shanghai Municipal Committee for the Advancement of Spiritual Civilization issued an initiative suggesting that when people have dinner, they should not forget to add chopsticks or a spoon for each dish.^21^ A total of 47.5% of rural residents always or frequently take Chinese herbal medicine to assist in health care, indicating that residents have high recognition of the preventive effect of Chinese herbal medicine.

The results of the regression analysis suggest that prevention and control interventions should be strengthened for females, people older than 30 years, farmers, people with poor economic conditions, those with no chronic diseases, people with no confirmed cases in their rural villages, and rural residents who have not experienced SARS. According to the results of the regression analysis, family economic level and knowledge were positive predictors of beliefs/attitudes. Knowledge, as a positive influencing factor of attitudes, is largely evidenced in the literature and fits with the KAP model. Rural residents with a lower economic level had significantly lower attitude scores, which might be ascribed to the fact that lower economic levels affected the formation of positive beliefs/attitudes, which was similar to the findings of other researchers conducting KAP studies on the influence of economic circumstances on attitudes. It was interesting to note that the SARS experience was a significant positive influencing factor of beliefs/attitudes in the univariate analysis (*P* < 0.01) but not in the multivariate analysis, suggesting that compared with other factors, the influence of prior experience on attitudes/beliefs might be relatively small. More studies are warranted in confirming the above findings, and residents with a lower economic and knowledge level should be the focus group for attitude interventions.

## DIFFICULTIES AND CHALLENGES

A total of 153 respondents answered that they experienced no difficulties or challenges, and 401 respondents may face one or more difficulties or challenges. The analysis and summary in this survey show that 72.4% of the residents had difficulties and challenges in epidemic prevention and control. The greatest difficulty is the lack of protective equipment (40.2%): rural residents, especially those in remote mountainous areas, purchase no masks, alcohol, or disinfectant, and rural medical workers, traffic police, and village staff on duty do not have protective clothing, such as isolation clothing and goggles. It is recommended that government departments give rural residents, especially those in remote mountainous areas, more front-line personnel when distributing protective materials. Furthermore, another issue is the inconvenience of travel (23.7%): vehicles are stopped and roads are obstructed (closed villages) and return trips are difficult (foreign residents are separated from their homes for 14 days at the destination), prompting government departments to humanely set up protective measures to close roads in closed villages and to adopt rational settings. Weak protection awareness (16.9%) is mainly reflected in the incubation period to conceal the illness. The elders do not wear masks outside, go outside during the epidemic period, and play mahjong, and family members do not wash their hands when they return home. These findings indicate that knowledge, attitude, and behavior scores are positively correlated. To solve the problem of persuasion ineffectiveness, villagers must have a good understanding of COVID-19 to encourage them to adopt a positive attitude and correct behavior and to prevent psychological problems (5.7%) (see Table 6). Long-term isolation and inactivity in homes will inevitably lead to boredom and panic about the disease. Villagers should be provided with appropriate psychological counseling or corresponding counseling channels during the epidemic so that they can report their mental disorders. At the same time, it is important to strengthen the rural network platform, appropriately reduce or exempt network traffic consumption, and let villagers chat with each other via video or play mahjong online, thereby reducing the chance of cross-infection.

## RESEARCH LIMITATIONS

This study conducted a rapid population survey in the early stages of the development of COVID-19. Using Internet survey tools, a sample size of 554 cases was obtained in a short period of time. The timeliness was good, and rural residents in the early stages of disease development could be quickly identified and their knowledge, attitude, and behavior could be assessed. A limitation of this study is the non-random sampling based on network invitation, which caused the sample to be under-represented in the rural population across the country. Furthermore, rural elderly people have limited access to mobile phones. Therefore, this part of the population may have failed to complete the questionnaire through WeChat. The information available to older people is limited, and the extrapolation of conclusions is therefore also limited.

Demographic characteristics are not representative of the population. The limitation of this study lies in the nonrandom sampling based on network invitation, and the sample characteristics are representative of individuals in the rural population, who have mobile phones with WeChat and skillfully use this software. To date, no scholars have investigated the proportion of elderly adults in rural areas who use mobile phones with WeChat in China. For example, some elderly people in rural areas have never gone to school, a large percentage of this population is illiterate, and the information available about them is limited.

## CONCLUSION

This study provided a preliminary discussion of the knowledge, beliefs/attitudes, behavior levels, and influencing factors of COVID-19 prevention and control in rural residents and the difficulties/challenges perceived during the implementation of prevention and control behaviors. Future research can focus on different aspects in different regions. Publicity and education on the prevention and control of COVID-19 can provide a reference basis, and scholars’ attention can be drawn to this research issue to expand the scope of investigation and populations in different countries and regions. This will help to comprehensively understand the current status of residents’ knowledge and behavior in the prevention and control of COVID-19. The influencing factors provide a more comprehensive and objective theoretical basis for further optimization of prevention and control strategies.

## Supplemental questionnaire

Supplemental materials
