# Supplementary material for: Knowledge, Beliefs/Attitudes, and Practices of Rural Residents in the Prevention and Control of COVID-19: An Online Questionnaire Survey
Source: Am J Trop Med Hyg. 2020 Oct 27;103(6):2357–67. doi: 10.4269/ajtmh.20-0314 (PMC7695081; doi:10.4269/ajtmh.20-0314)
Supplement: Supplementary file 1 [file tpmd200314.SD1.pdf]

Supplemental Questionnaire

Prevention and control of COVID-19

1. Incubation period **Fill in blank** (3-7days, maximum 14 days: 1 point)
2. Source of infection **Multiple choice**

- Confirmed COVID-19 patients with symptoms (1 point)
- People who have no symptoms but are in the incubation phase of COVID-19 (1 point)
- fever, cough and phlegm is the source of infection (1 point)
- Not clear (0 point)

3. Route of transmission **Multiple choice**

- Respiratory tract infection (1 point)
- Contact transmission (1 point)
- Alimentary tract transmission (1 point)
- Not clear (0 point)

4. Medical observation time **Fill in blank** (14days, 1 point)
5. Close contact **Multiple choice**

- Persons who had close contact with a confirmed case from 3 days before onset to after onset (1 point)
- Persons who had close contact with a suspected case from 3 days before onset to after onset (1 point)
- Close contact with asymptomatic persons who have tested positive for the virus 3 days prior to the positive test (1 point)
- Not clear (0 point)

6. Major symptom **Multiple choice**

- With fever (1 point)
- fatigue (1 point)
- dry cough as the main manifestations (1 point)
- a few accompanied by nasal congestion, runny nose, diarrhea and other symptoms (1 point)
- To diarrhea, nausea and vomiting and other symptoms of the digestive system (1 point)
- Not clear (0 point)

7. Nearby designated hospital **Single choice**

- don’t know (0 point)
- partly know (1 point)
- know (2 point)

8. Susceptible people **Multiple choice**

- Young people (1 point)
- Children (1 point)
- Middle aged people (1 point)
- Old people (1 point)
- Pregnant (1 point)
- Not clear (0 point)

9. Classification of infectious disease **Fill in blank** (Category B infectious disease, 1 point)
10. Transmission rate (1 person can infect 2-3 people, 1 point) and doubling time (6-7 days, 1 point) **Fill in blank**
11. Prevention and control measures **(Multiple choice)**

- Wear masks when going out (1 point)
- Avoid going to crowded places (1 point)
- Not attend party/gathering (1 point)
- Avoid going out (1 point)
- Wash hands while going back home, before meals, after using the toilet, or contacting with dirty and contaminated items (1 point)
- Open window to improve air circulation (1 point)
- Not eat wild animals (1 point)
- Avoid taking public transportation (1 point)
- Isolate at home and see doctor if have contact history or symptoms (1 point)
- Cover nose and mouth when cough or sneeze (1 point)
- Use disposable paper napkin for access to public facilities such as elevator buttons, and door handles (1 point)
- Avoid going to live poultry market (1 point)
- Take balanced nutrition diet (1 point)
- Keep a good sleep (1 point)

- Use serving chopsticks or separate meals (1 point)
- Drink more water (1 point)
- Take temperature (1 point)
- Keep a good mood (1 point)
- Hang the worn clothes on the balcony or other ventilated place (1 point)
- Sterilize exposed parts and clothing with alcohol or chlorine-containing disinfectant when returning home (1 point)
- Wipe furniture surface and household things with alcohol or chlorine-containing disinfectant (1 point)
- Wear warm to prevent catching a cold (1 point)
- Exercise, such as running on treadmill, indoor yoga, and tai chi (1 point)
- Take Chinese herbal medicines for nourishing yin, nourishing vitality, tonifying spleen, and moistening lung (1 point)
